# Variation in genomic islands contribute to genome plasticity in *cupriavidus metallidurans*

**DOI:** 10.1186/1471-2164-13-111

**Published:** 2012-03-23

**Authors:** Rob Van Houdt, Pieter Monsieurs, Kristel Mijnendonckx, Ann Provoost, Ann Janssen, Max Mergeay, Natalie Leys

**Affiliations:** 1 Unit of Microbiology, Belgian Nuclear Research Centre (SCK•CEN), B-2400 Mol, Belgium; 2 Laboratory of Food and Environmental Microbiology, Université catholique de Louvain, B-1348 Louvain-la-Neuve, Belgium

## Abstract

**Background:**

Different *Cupriavidus metallidurans *strains isolated from metal-contaminated and other anthropogenic environments were genotypically and phenotypically compared with *C. metallidurans *type strain CH34. The latter is well-studied for its resistance to a wide range of metals, which is carried for a substantial part by its two megaplasmids pMOL28 and pMOL30.

**Results:**

Comparative genomic hybridization (CGH) indicated that the extensive arsenal of determinants involved in metal resistance was well conserved among the different *C. metallidurans *strains. Contrary, the mobile genetic elements identified in type strain CH34 were not present in all strains but clearly showed a pattern, although, not directly related to a particular biotope nor location (geographical). One group of strains carried almost all mobile genetic elements, while these were much less abundant in the second group. This occurrence was also reflected in their ability to degrade toluene and grow autotrophically on hydrogen gas and carbon dioxide, which are two traits linked to separate genomic islands of the Tn*4371*-family. In addition, the clear pattern of genomic islands distribution allowed to identify new putative genomic islands on chromosome 1 and 2 of *C. metallidurans *CH34.

**Conclusions:**

Metal resistance determinants are shared by all *C. metallidurans *strains and their occurrence is apparently irrespective of the strain's isolation type and place. *Cupriavidus metallidurans *strains do display substantial differences in the diversity and size of their mobile gene pool, which may be extensive in some (including the type strain) while marginal in others.

## Background

Metals are common in our environment and diet. Rocks and soils are the principal and natural sources of metals in the environment. These sources lead to natural background levels in soils, sediments, waters and organisms that are supplemented by many human activities like agriculture (fertilizers, manure and pesticides) and industrial activities (petrochemical, extractive, metallurgic). Some metals are essential trace elements, however, many are toxic to organisms. The essential elements are acquired from the environment depending on the necessities and their uptake is strictly controlled by homeostasis. Failure of these regulation mechanisms due to metal deficiency or toxicity (excess) results in harmful effects on the organism. Mechanisms to resist metals are abundant and widespread in bacteria, with resistance determinants occurring in a few percent in pristine environments to in nearly all isolates in heavily polluted environments [1].

*Cupriavidus metallidurans *is a species characterized by multiple metal-resistances [2-4]. For *C. metallidurans *type strain CH34 a substantial part of the metal resistance mechanisms are carried by its two megaplasmids pMOL28 and pMOL30 [5,6]. However, next to these specialized plasmids also chromosomally-encoded metal responsive gene clusters have been identified [7]. The *C. metallidurans *CH34 genome [8] hosts in addition a large diversity of mobile genetic elements (MGEs) including genomic islands (GIs), integrative and conjugative elements, transposons and Insertion Sequence (IS) elements [9,10]. Both the number and diversity of genes related to MGEs is larger in type strain CH34 than in related strains from other *Cupriavidus *and *Ralstonia *genera [8,9]. Multiple GIs, in particular on both plasmids, contain genes for heavy metal resistance [6,9]. Its capacity to degrade toluene, to fix carbon dioxide and to oxidize hydrogen is located on Tn*4371*-like integrative and conjugative elements [9,11,12].

*C. metallidurans *strains were often isolated from industrial sites linked to mining-, metallurgical-, and chemical industries [2,13,14]. Next to this, *C. metallidurans *strains were isolated from environments not typified by metal contamination like, for instance, from different spacecraft-related environments [15-19], from patients with cystic fibrosis [20] or even as the causative agent of an invasive human infection [21].

In this study, we aimed to gain insight in the dispersion and horizontal transfer of genes and the evolutionary forces shaping this species. Therefore, whole-genome oligonucleotide DNA microarrays based on the genome of CH34 were used to compare 16 *C. metallidurans *strains isolated from diverse metal-contaminated biotopes, from other anthropogenic environments and from human cerebrospinal fluid with type strain CH34.

## Results

### General comparison

Comparative whole-genome hybridization (CGH) was applied to compare sixteen *C. metallidurans *strains (Table 1) with type strain CH34 and showed that all strains shared a core of 3387 coding sequences (CDS), which represents 54.6% of the 6205 oligonucleotide probes present on the CH34 microarray. This common gene pool represented 58.2%, 53.7%, 16.4% and 31.0% of chromosome 1 (CHR1), chromosome 2 (CHR2), pMOL28 and pMOL30, respectively. Thus, although the main replicon (CHR1) carries most of the housekeeping genes [8], only a slightly higher percentage of CDS is conserved in CHR1 compared to CHR2. This is different when comparing the sequenced *Cupriavidus *species (*C. metallidurans, C. eutrophus, C. pinatubonensis, C. taiwanensis*), for which the percentage of common genes is much larger for CHR1 than for CHR2 [8].

**Table 1:** Strains used in this study

Strain	Isolation site	Isolation place	Reference
AS39	Mine tailings	Likasi-Sud, Congo	[14]
AS167	Mine tailings	Likasi-Sud, Congo	[13]
AS168	Mine tailings	Likasi-Sud, Congo	[14]
KT01	Wastewater treatment plant	Göttingen, Germany	[22]
KT02	Wastewater treatment plant	Göttingen, Germany	[23]
KT21	Wastewater treatment plant	Göttingen, Germany	[22]
SV661	Non-ferrous industry	Beerse, Belgium	[14]
CH34^T^	Decantation tank, zinc factory	Liège, Belgium	[24]
CH42	Polluted sediments, zinc factory	Liège, Belgium	[13]
CH79	Polluted sediments, zinc factory	Liège, Belgium	[13]
31A	Galvanization tank, metal factory	Holzminden, Germany	[23]
NE12	Cleanroom Kennedy Space Center	Florida, USA	[25]
NA1	Water storage system	International Space Station	[26]
NA2	Contingency water container	International Space Station	[26]
NA4	Water recovery system	International Space Station	[26]
43015	Human cerebrospinal fluid	Göteborg, Sweden	CCUG*
45957	Pharmaceutical industry	Sweden	CCUG

*Culture Collection, University of Göteborg, Sweden

Within this conserved group 2760 CDS were assigned to a Cluster of Orthologous Groups (COG). The most abundant COG functional categories were function unknown (Category S; 12.4%), amino acid transport and metabolism (Cat. E; 9.3%), general function prediction only (Cat. R; 9.0%), transcription (Cat. K; 8.5%) and energy production and conversion (Cat. C; 8.4%), respectively.

**Figure 1:**
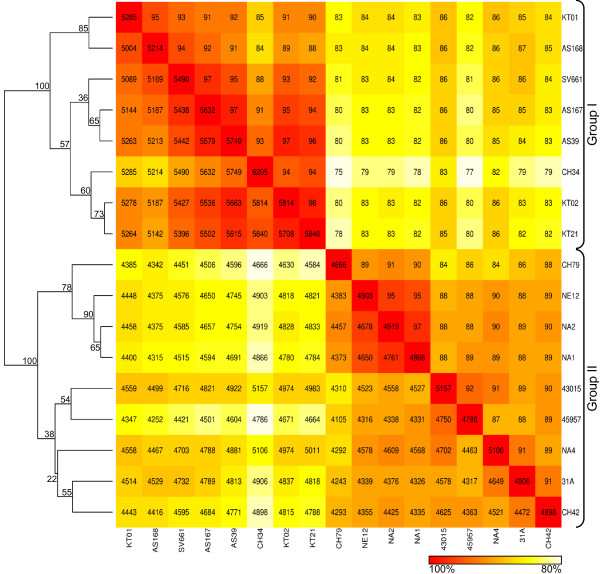
**Graphical representation and clustering analysis of *C. metallidurans *strains**. Hierarchically (complete-linkage) clustered heat map based on CGH results of 17 different *C. metallidurans *strains to a whole-genome oligonucleotide DNA microarray of type strain CH34. The numbers indicated on the heat map are the total number (below diagonal) and percentage (above diagonal) of genes shared between two corresponding strains. The numbers on the diagonal represent the maximal number of genes within one species that gave a detectable signal on the DNA microarray. Bootstrap values (%) from 1,000 times resampling are shown at each dendrogram node.

Cluster analysis based on the pairwise number of overlapping orthologs indicated that all strains can clearly be clustered into two main groups (Figure 1). The strains isolated from the mine tailings in Congo (AS39, AS167 and AS168) grouped together with SV661 isolated from a metal factory in Belgium and with KT01 from a wastewater treatment plant in Germany. This cluster grouped together with a cluster comprising type strain CH34 and the other two isolates KT02 and KT21 from the wastewater treatment plant in Germany forming group I (Figure 1). Group II included the strains isolated from the spacecraft-related environments (NE12, NA1, NA2 and NA4), as well as 31A (Germany) and CH42 (Belgium) from metal factories, the clinical strain 43015 (Sweden), and strain 45957 from pharmaceutics (Sweden). Interestingly, type strain CH34 did not group with the two other strains (CH42 and CH79) isolated from the same site in Belgium.

### Mobile genetic elements

**Figure 2:**
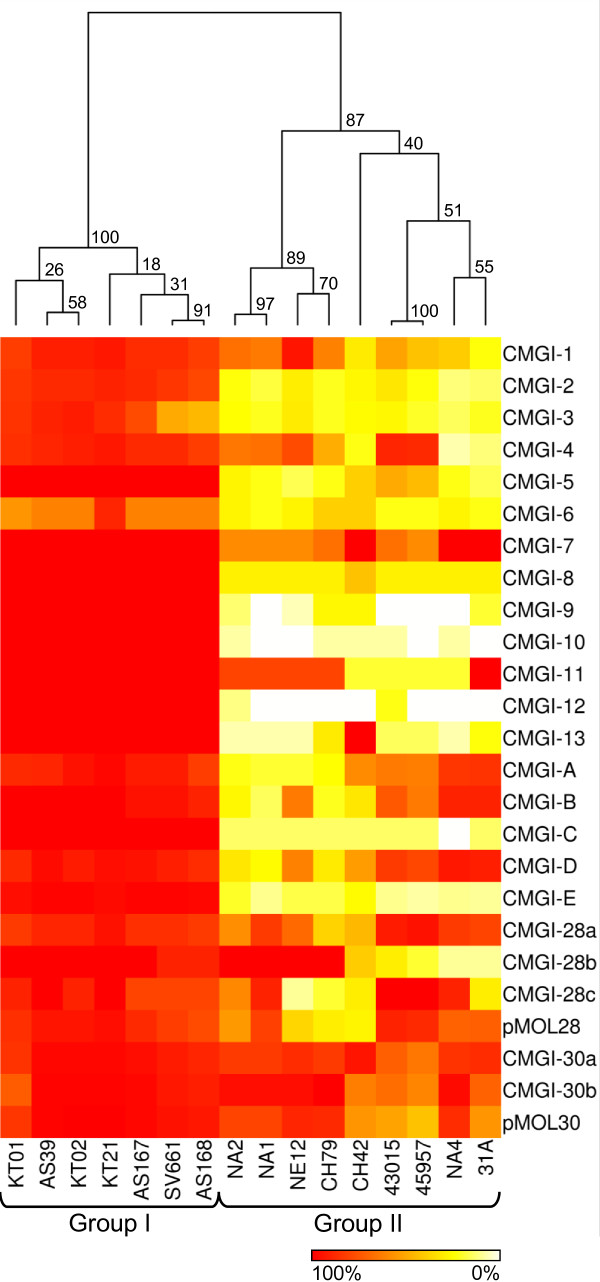
**Graphical representation and clustering analysis of MGEs in *C. metallidurans *strains**. Hierarchically (complete-linkage) clustered heat map based on CGH results related to plasmids, previously and newly identified genomic islands of 16 different *C. metallidurans *strains to a whole-genome oligonucleotide DNA microarray of type strain CH34. Bootstrap values (%) from 1,000 times resampling are shown at each dendrogram node.

All strains (except CH42) carried one or more megaplasmids (Additional file 1: Figure S1). Concordantly, a good overall conservation of pMOL28 and pMOL30 genes was observed. CGH indicated that for pMOL30 genes in general between 80 and 99% of the probes showed a positive hybridization signal except for CH42 (55.6%), 31A (55.6%), 43015 (51.4%) and 45957 (42.6%) (Figure 2, Table 2). For KT02, 99% or 214 out of 216 gave a positive hybridization signal. For CH42, 31A, 43015 and 45957, a higher percentage of positive hybridization signals was observed for the genomic islands CMGI-30a and CMGI-30b carried by pMOL30 than for the plasmid backbone, indicating that the megaplasmid(s) in these strains probably do not carry a backbone similar to pMOL30. The pMOL28 genes are less conserved than those from pMOL30 but still in general between 71 and 95% with the highest number of positive CGH signals for KT21 (95%) and KT02 (94%) (Figure 2, Table 2). Conservation below 70% was observed for CH42 (29.6%), CH79 (31.6%), NE12 (38.8%) and NA2 (55.9%). More positive hybridization signals were found for the GIs on pMOL28 than the plasmid backbone, indicating that their megaplasmids probably do not carry a backbone similar to pMOL28. Concordantly, strains lacking either the pMOL28 backbone (CH79, NE12 and NA2), the pMOL30 backbone (43015 and 45957) or both (CH42) have only one or no megaplasmid (Additional file 1: Figure S1). Strain 31A, which carries two megaplasmids pTOM8 and pTOM9 [27], showed only a positive hybridization signal with the pMOL28 backbone, indicating that one of both has a backbone related to pMOL28 while the other has a backbone unrelated to pMOL30.

**Table 2:** Relative occurrence of *C. metallidurans *CH34 genomic islands and plasmids in *C. metallidurans *strains as indicated by CGH*

CMGI	AS39	AS167	AS168	KT01	KT02	KT21	SV661	CH42	CH79	31A	NE12	NA1	NA2	NA4	43015	45957
CMGI-1	90.8	87.5	81.7	81.7	90.8	91.7	86.7	29.2	62.5	23.3	92.5	63.3	65.8	37.5	50.0	41.7
CMGI-2	86.6	86.6	78.6	83.0	86.6	89.3	83.0	27.7	22.3	15.2	30.4	18.8	24.1	12.5	32.1	25.0
CMGI-3	89.5	77.9	47.7	84.9	91.9	87.2	51.2	26.7	22.1	20.9	30.2	26.7	29.1	20.9	30.2	24.4
CMGI-4	88.7	86.8	81.1	84.9	90.6	92.5	86.8	22.6	47.2	13.2	77.4	66.0	64.2	7.5	88.7	86.8
CMGI-5	**100**	**100**	**100**	**100**	**100**	**100**	**100**	38.9	22.2	16.7	16.7	22.2	27.8	22.2	50.4	44.4
CMGI-6	46.2	46.2	46.2	38.5	46.2	84.6	46.2	23.1	30.8	15.4	7.7	0	7.7	7.7	0	0
CMGI-7	**100**	**100**	**100**	**100**	**100**	**100**	**100**	**100**	69.2	**100**	61.5	53.8	61.5	**100**	69.2	53.8
CMGI-8	**100**	**100**	**100**	**100**	**100**	**100**	**100**	42.9	28.6	28.6	28.6	28.6	28.6	28.6	28.6	28.6
CMGI-9	**100**	**100**	**100**	**100**	**100**	**100**	**100**	20.0	20.0	13.3	6.7	0	13.3	0	0	0
CMGI-10	**100**	**100**	**100**	**100**	**100**	**100**	**100**	7.1	7.1	0	0	0	7.1	7.1	7.1	0
CMGI-11	**100**	**100**	**100**	**100**	**100**	**100**	**100**	14.3	57.1	**100**	57.1	57.1	57.1	28.6	28.6	28.6
CMGI-12^§^	**100**	**100**	**100**	**100**	**100**	**100**	**100**	0	0	0	0	0	11.1	0	22.2	0
CMGI-13^§^	**100**	**100**	**100**	**100**	**100**	**100**	**100**	**100**	31.3	25.0	6.3	6.3	12.5	12.5	18.8	18.8
CMGI-A^§^	89.0	91.8	82.2	87.7	94.5	97.3	91.8	57.5	24.7	84.9	19.2	19.2	21.9	83.6	63.0	61.6
CMGI-B^§^	**100**	95.2	85.7	**100**	**100**	**100**	95.2	28.6	19.0	85.7	61.9	14.3	23.8	85.7	71.4	61.9
CMGI-C^§^	**100**	**100**	**100**	**100**	**100**	**100**	**100**	14.3	14.3	14.3	14.3	14.3	14.3	0	14.3	14.3
CMGI-D^§^	96.2	95.2	86.5	87.5	91.3	95.2	91.3	52.9	31.7	91.3	63.5	26.9	32.7	95.2	82.7	77.9
CMGI-E^§^	99.0	98.0	98.0	96.0	99.0	97.0	99.0	25.7	17.8	9.9	17.8	10.9	20.8	10.9	10.9	8.9
CMGI-28a	91.1	88.9	86.7	86.7	91.1	95.6	88.9	53.3	44.4	73.3	71.1	86.7	66.7	86.7	93.3	95.6
CMGI-28b	**100**	**100**	83.3	91.7	**100**	**100**	91.7	33.3	**100**	8.3	**100**	**100**	91.7	8.3	25.0	16.7
CMGI-28c	**100**	83.3	75.0	83.3	83.3	91.7	83.3	25.0	16.7	33.3	8.3	75.0	50.0	75.0	91.7	91.7
pMOL28	93.4	87.5	77.6	86.2	94.1	95.4	82.2	29.6	31.6	72.4	38.8	81.6	55.9	71.7	90.1	88.2
CMGI-30a	98.4	96.8	91.9	88.7	98.4	98.4	93.5	93.5	83.9	83.9	88.7	85.5	85.5	87.1	77.4	72.6
CMGI-30b	98.8	97.6	90.4	73.5	98.8	98.8	92.8	61.4	**100**	71.1	95.2	95.2	95.2	96.4	66.3	59.0
pMOL30	98.6	97.2	92.6	83.3	99.1	99.1	94.4	55.6	88.0	55.6	88.4	80.1	80.1	86.6	51.4	42.6

*Percentage of genes related to a particular genomic island (CMGI) or plasmid (pMOL) displaying a positive hybridization signal by CGH. ^§^Genomic islands identified in this study.

The strong conservation of the GIs on pMOL28 (especially CMGI-28a) and pMOL30, which carry all the metal resistance determinants of these plasmids, already indicated a high conservation of the metal responsive clusters (see below).

Next to the megaplasmids and their GIs, CH34 carries 11 previously identified GIs on chromosome 1 [6,9]. Based on CGH data analysis for the presence of GIs, the strains could again be divided into two main groups. One group (KT01, KT02, KT21, AS39, AS167, AS168 and SV661) carried almost all GIs identified in CH34, while these GIs were much less abundant in the second group (31A, CH42, CH79, NE12, NA1, NA2, NA4, 43015 and 45957) (Table 2, Figure 2). This clustering (Figure 2) resembled the clustering based on all oligonucleotide probes (Figure 1), which indicated that the presence of GIs is the main source of divergence in these strains. At least 11 strains carried CMGI-1 (or a large part of it). CMGI-1 of CH34 is a 109 kb GI of the PAGI-2 family and is almost 100% identical to PAGI-2 C of *P. aeruginosa *clone C isolated from a cystic fibrosis patient [9,28]. These data are in agreement with the analysis of the KT and CH strains by Klockgether et al. [28]. The relative occurrence also indicated that CMGI-5 and CMGI-7 to CMGI-11 were highly conserved when present. CMGI-2 to CMGI-4 belong to the Tn*4371 *family [9,11,12,29] and were previously designated ICE_Tn*4371*_6054, ICE_Tn*4371*_6055 and ΔICE_Tn*4371*_6056, respectively [29]. CMGI-2 (ICE_Tn*4371*_6054) and CMGI-3 (ICE_Tn*4371*_6055) are responsible for CH34's ability to grow on aromatic compounds and to fix carbon dioxide, respectively. The presence of these GIs in the *C. metallidurans *strains is in accordance with their ability to degrade toluene or to grow on hydrogen gas and carbon dioxide (Additional file 2: Table S1). Except for NE12 that apparently lacks the genes involved in degradation of aromatic compounds but is able to degrade toluene and vice versa for AS39 that apparently carries the genes but lacked degradation ability. Therefore, NE12 putatively carries other functional genes, while for AS39 functionality is probably lost. Finally, the presence of other Tn*4371*-like elements in these strains can not be excluded. Especially since all strains except CH42, 31A and NA4 displayed good conservation of the partial CMGI-4 (ΔICE_Tn*4371*_6056), which lacks the transfer module [9], and displayed positive hybridization signals related to the transfer module of CMGI-2.

The mosaic structure of the second chromosome of *C. metallidurans *CH34, but also other *Cupriavidus, Ralstonia *and *Burkholderia *strains, made it complicated to clearly identify GIs on this replicon. Consequently, no GIs on chromosome 2 of *C. metallidurans *CH34 were defined in a previous study [9]. Here we took advantage of the clear pattern of GI distribution in chromosome 1 over the 16 different *C. metallidurans *strains to scan chromosome 2 for similar patterns (Additional file 3: Figure S2), which could be an indication for the presence of a genomic island. Five different GIs could be identified (Tables 2 and 3, Figure 2). Interestingly, in CH34 both CMGI-B and CMGI-D flank a copy of the Tn*6050 *transposon. It was previously shown that in CH34 a chromosomal inversion occurred by recombination between the pair of Tn*6050 *transposons [9]. Therefore, CMGI-B and CMGI-D are in fact two parts of the same genomic island of 160.7 kb with at one extremity multiple genes coding for phage-related proteins (associated to phages of *Ralstonia solanacearum*) and at the other extremity a cluster of 22 genes in synteny with *R. solanacearum*. Accordingly, the relative occurrence of CMGI-B was comparable to that of CMGI-D for each strain, and both were conserved (> 60%) in 13 strains (Table 2, Figure 2). In CH34, the 120 kb CMGI-E carries at one extremity an IS*Rme3*, three Tn*7*-related genes (*tnsABC*) and an IS*Rme11 *inserted into *tnsC*. In fact a very small gene cluster (located approximately 1.89 Mb upstream or +/- 687 kb downstream) in chromosome 2 carried a IS*Rme3 *and two Tn*7*-related genes (*tnsD1 tnsD2*). Furthermore, for each strain the presence of these genes coincides with the presence of *tnsABC*. To determine the relation between these distant Tn*7*-related genes, the STRING database (version 8.3; http://string.embl.de) was used to find genomes where these genes occur as immediate neighbors. This identified with high confidence (based on STRING parameters) orthologous groups of TnsABCD2 in *Anabaena variabilis *and of TnsABCD1 in *Hahella chejuensis, Shigella sonnei *and *Idiomarina loihiensis*. Additional BLASTP searches indicated the presence of this cluster also in strains more closely related to CH34 such as *Burkholderia phymatum *and *Herminiimonas arsenicoxydans *(both β-Proteobacteria). These results evidenced that a putative chromosomal inversion of a very large region (+/- 687 kb) occurred in CH34 by recombination between the pair of IS*Rme3 *elements. In fact, before inversion the region formed a genomic island with a set of Tn*7*-encoded proteins (TnsABCD1D2) at one extremity and accessory genes putatively involved in the degradation of aromatic compounds.

**Table 3:** Newly identified putative genomic islands on chromosome 1 and chromosome 2 of *C. metallidurans *CH34

GI	Replicon	Size (kb)	Coordinates	Features
CMGI-12	CHR1	9.1	2,895,950-2,905,048	Direct repeats (3' end sequence of a boundary tRNA gene), genes coding for hypothetical proteins
CMGI-13	CHR1	15.9	2,957,898-2,973,796	Genes involved in polysaccharide biosynthesis
CMGI-A	CHR2	87.1	813,173-900,292	IS*Rme1 *at one extremity
CMGI-B	CHR2	19.5	1,075,185-1,094,655	Multiple genes coding for phage-related proteins (associated to phages of *Ralstonia* *solanacearum*)
CMGI-C	CHR2	7.1	1,160,750-1,167,881	Fragment of tyrosine-based site-specific recombinase at one extremity, direct repeats (41 bp), gene coding for mannose-binding lectin
CMGI-D	CHR2	141.2	1,217,667-1,358,908	At one extremity 22 genes in synteny with *R*. *solanacearum*
CMGI-E	CHR2	120.2	2,202,142-2,322,293	Tn*7*-related genes at one extremity, genes putatively involved in degradation of aromatic compounds

The above described observations also encouraged us to scrutinize once more chromosome 1 of CH34 for putative genomic islands (Additional file 4: Figure S3). In addition to the previously identified islands [9], at least two other putative GIs could be identified (Tables 2 and 3, Figure 2). CMGI-12 carries genes coding for hypothetical proteins and is flanked by direct repeats (3' end sequence of a boundary tRNA gene). CMGI-13 carries genes involved in polysaccharide biosynthesis, however, no genes putatively involved in mobility could be identified.

The occurrence of insertion sequences in the different *C. metallidurans *strains followed the same trend as that of the GIs. Based on CGH the same two main clusters could be derived. One group (KT01, KT02, KT21, AS39, AS168, AS169 and SV661) displayed positive hybridization signals for 90 to 98% of the 42 probes related to IS elements, while the second group (31A, CH42, CH79, NE12, NA1, NA2, NA4, 43015 and 45957) only displayed 43 to 62% positive signals (data not shown).

The occurrence of transposon Tn*6048 *displayed the same pattern as for the IS elements and GIs, however, Tn*6049 *and the mercury transposons (Tn*4378 *and Tn*4380*) appear to be present in all strains (data not shown).

### Heavy metal resistance genes

The presence of all gene clusters, which are identified in CH34 as involved in heavy metal detoxification, was evaluated in the genomes of the other strains (Table 4). Almost all metal responsive clusters where found in the other isolates, however, some clusters did not fully correspond to those from CH34. The metal resistance arsenal of CH34 is most conserved in KT21 followed by KT02, AS39, AS167 and SV661. These strains also grouped into a separate cluster based on total genome comparison (Figure 1).

**Table 4:** Occurrence of *C. metallidurans *CH34 metal resistance gene clusters in *C. metallidurans *strains as indicated by CGH^a, b^

**Gene cluster in CH34**^**c, d**^	AS39	AS167	AS168	KT01	KT02	KT21	SV661	CH42	CH79	31A	NE12	NA1	NA2	NA4	43015	45957
*cdfX, pbrR_2_cadA pbrC_2_*,								*XAC_2_*	*XAC_2_*	*XAC_2_*		*XAC_2_*	*XAC_2_*	*XAC_2_*	*XAC_2_*	*XAC_2_*
*pbrR_3_*																
*pbrU_b_*|*U_a_TR pbrABCD*				*R*					*UbUa*						*UbRAD*	*UbRABCD*
*merRTPA'A″*			*R*	*R*				*R*	*R*	*R*		*RPA*	*RPA*	*R*	*R*	*RA*
*merRTPADE urf-2*																
*merRTPADE urf-2*																
*merRT*Δ*P*								*RT*Δ*P*	*RT*Δ*P*	*RT*Δ*P*	*RT*Δ*P*	*RT*Δ*P*	*RT*Δ*P*	*RT*Δ*P*	*RT*Δ*P*	*RT*Δ*P*
*ChrBAF*			*F*	*F*					*BF*		*BF*	*BF*	*BF*			
*chrIBACEFONPYZ*								*IBACEOY*	*IBACEFOY*		*Z*		*BO*		*O*	
*cusDCBAF*			*D*	*D*			*D*	*DB*	*D*	*D*			*D*			*D*
*silDCBA cus*Δ*F*				*C*											*F*	*F*
*copSRABCD*	*S*	*S*	*SC*	*SC*	*C*	*C*	*SC*	*SC*	*C*	*SC*	*C*	*C*	*C*		*SC*	*C*
*copV-W*(*)			*IJF*	*KCIJFOH*			*FH*									
*czcLRS ubiG czcB_a_*|*B_b_CI*			*I*	*I*					*I*						*I*	*I*
*zntA*																
*czcMNICBADRSEJ ompP*			*B*	*BS*					*ompP*			*S*	*S ompP*	*S*		
*czcP*												*ompP*				
*nimBA_a_*|*A_b_C*	*C*	*C*	*BC*	*BC*	*C*		*C*	*C*	*BC*	*BC*					*C*	*C*
*cnrYXHCBAT*	*HC*	*HC*	*YHC*	*HC*	*HC*	*C*	*HC*	*YXHCBA*	*YXHCBAT*	*C*	*HC*	*HC*	*YXHCA*	*C*	*C*	
*nccCB″B'A nreB*			*CB'*	*CB*			*B'*	*CB"B'A*			*B"*	*B"*			*CB'*	*CB"B'A*
*arsPHC_1_BC_2_IRM*																
*cupRAC*				*R*					*R*							
*agrCBARS*		*S*	*S*									*B*				
*hmzRS hmzB*Δ*A*	*RB*	*RB*	*RBA*	*RBA*	*R*	*R*	*B*	*RSBΔA*	*RSBΔA*	*RSBΔA*	*R*	*R*	*B*	*RSBΔA*	*R*	*R*
*hmvCB*Δ*A*				*CB*					*C*		*BA*	*BA*	*BA*	*BA*		*A*
*zniABCSR znePRSCAB*^(#)^	*C^i^*	*C^i^*	*C^i^*	*C^i^*	*C^i^*	*C^i^*	*C^i^*	*C^i^*	*C^i^*	*C^i^*	*C^i^*	*C^i^C^e^*	*C^i^C^e^*	*C^i^*		
*hmyCB|hmyA*			*C*	*C*							*A*	*A*				

^a^Only genes that did not display positive hybridization signals are shown. ^b^See ref. [8] for the extensive and descriptive table with metal detoxifications in *C. metallidurans *CH34. ^c^Underlined genes are regulators; ^d^Insertion (IS or Tn), frameshift and truncation are indicated by (*|*), (','') and (Δ), respectively. *Complete cluster is *copVTKMNSRABCDIJGFOLQHEW*; ^#^*zniC *(*C*^i^) and *zneC *(*C^e^*).

A noticeable phenotypic difference was observed for nickel, chromate and lead resistance compared to CH34 (Additional file 2: Table S2). For most isolates, including CH34, the maximum tolerable nickel concentration is around 4 mM, however, this concentration is much higher for NA4, KT01, KT02, KT21 and 31A (Additional file 2: Table S2). For strains 31A and KT02, it has been shown that the *nccYXHCBAN *locus is responsible for the resistance to 40 mM nickel [30]. The *nccCBA *locus in CH34 bears a frameshift in *nccB *indicating that the *ncc *genes probably are not functional [5]. The pMOL28-encoded chromate cluster (*chrIchrBACEFONPYZ*) is almost completely absent in strains CH42 and CH79 (Table 4). Concordantly, the maximum tolerable chromate concentration for these strains is around 0.1 mM, while this concentration is around 0.2 mM for the other strains (Additional file 2: Table S2). Similarly, the pMOL30-encoded lead cluster (*pbrABCD*) is almost completely absent in strains 43015 and 45957 (Table 4), resulting in a lower MTC compared to the other strains (Additional file 2: Table S2).

Strain CH34 carries several metal resistance genes that are putatively inactivated by frame shift mutation (like *nccB*, see above), truncation or insertion by an IS element or transposon (Table 4). The presence of three different insertions was evaluated for all isolates, specifically (*i*) insertion of IS*1088 *between *hmyA *and *hmyB *(coding for part of a resistance-nodulation-cell division (RND) transporter), (*ii*) insertion of IS*Rme3 *in *nimA *(coding part of system), and (*iii*) insertion of Tn*6049 *in *pbrU *(encoding a Major Facilitator Superfamily permease).

The presence of these insertions followed the same trend as the occurrence of insertion sequences (described above). Insertions of IS*1088*, IS*Rme3 *and Tn*6049 *were present in strains KT01, KT02, KT21, AS39, AS167, AS168 and SV661 but not in strains CH42, CH79, 31A, NE12, NA1, NA2, NA4, 43015 and 45957 (data not shown). The occurrence of an insertion in *nimBAC*, which codes for a RND transporter putatively involved in the resistance to Ni^2+ ^and Co^2+ ^[3,8], does not clearly influence the MTC of nickel or cobalt, however, the presence of other (plasmid-encoded) Ni^2+ ^and Co^2+ ^resistance determinants could mask this. Similarly, the occurrence of an insertion in *pbrU *is not univocally related to a lower MTC (Additional file 2: Table S2). Finally, it should be noted that potentially additional metal resistance determinants could be present in the other strains, which are undetectable with a CGH array based on the CH34 genome.

### Genes encoding sigma factors and small stress responsive proteins

No less than 18 different sigma factors were identified in *C. metallidurans *CH34, which enable specific binding of RNA polymerase to promoters, and are activated in response to different environmental changes [8]. Generally, the number and diversity of sigma factor genes in a certain genome relates to the environmental variation allowing growth [31], which thus indicates that *C. metallidurans *CH34 is able to respond to a broad range of environmental changes. CGH showed for all *C. metallidurans *strains positive signals for 12 sigma factors (Additional file 2: Table S3). Hybridization signals below the threshold were found for the housekeeping sigma-70 factors RpoD1 and RpoD2 for strain AS168, KT01 and CH79, and NE12, NA1 and 45957, respectively. Sigma factor CnrH carried by plasmid pMOL28 and encoded by the *cnr *gene cluster, which is involved in nickel resistance, was only observed for KT21, 31A, NA4, 43015 and 45957. Next to these, differences were observed for RpoR (for strains KT01, CH79, AS39, AS167, AS168, 31A, SV661), RpoP (for strains CH79, NE12, NA1, NA2, 43015 and 45957) and RpoJ (for strain NA4).

Another interesting group of genes in CH34 consists of 19 homologous genes encoding for putative small (between 69 and 165 amino acids) stress responsive proteins in CH34, which are likely to be secreted since all have a distinctive signal peptide and are apparently only found in *Cupriavidus *and *Ralstonia *species [8]. Ten of these genes were found to be induced in response to different heavy metals [7] like the pMOL30-encoded *copQ *[32] and *czcJ *[33], while three others were induced by hydrogen peroxide (Saiful Islam Muhammed, pers. comm.). CGH indicated that these genes are well conserved among *C. metallidurans *strains (Additional file 2: Table S4).

## Discussion

Comparative whole-genome hybridization (CGH) was used to compare sixteen *C. metallidurans *strains isolated from different biotopes with type strain CH34. Although the nature of these sites differs, ranging from pharmaceutical and space industry to metal mining and metal industries, waste treatment plants and even human infection, at least the oligotrophic aspect is common.

The global comparison indicated that while chromosome 1 is the ancestral replicon of the *Cupriavidus *genus, chromosome 2 appears to be more specific to and conserved within the *C. metallidurans *species. These results are in agreement with the study of Bavishi and colleagues [34], which indicated that chromosome 2 evolves faster than chromosome 1, leading to different conservation on inter- and intraspecies level. This global comparison also indicated that strain CH34 is more closely related to strains isolated from Congo than with the two other strains (CH42 and CH79) isolated in Belgium. This supports an old assumption that strain CH34 was transported into Belgium by the import of ores from Congo, which was a Belgian colony from 1908 until 1960.

The incidence of MGEs showed a clear pattern as well as evident phenotypes carried by them like the degradation of toluene or the ability to grow on H_2 _and CO_2_. No clear correlation was found between the occurrence of some MGEs and isolation site characteristics and location (geographic). However, it was apparent that strains carried either most of the MGEs or only a few. Interestingly, strains isolated from more hygienic settings like clinical, pharmaceutical or spacecraft environments carried almost no MGEs contrary to strains isolated from sites related to raw materials or environmental sources. This could indicate that either acquisition or loss of these MGEs was advantageous at one point enabling their spread in numerous environments and locations. For example, the MGEs related to hydrogenotrophy and degradation of toluene may recall to the volcanic origin [35-37] and such MGEs may have been dispensable and lost in technologized environments. Whereas MGEs with a putative link (however without direct evidence at this time) to opportunistic infection were maintained, such as CMGI-1, which is almost identical to the PAGI-2 C island identified in *Pseudomonas aeruginosa *clone C (isolated from a cystic fibrosis patient), and CMGI-11, which carries fimbrial genes.

The clear presence or absence of a multitude of MGEs in this group of *C. metallidurans *strains allowed us to further scrutinize the genome of CH34 for MGEs, especially in the second chromosome. At least five additional regions could be identified with one island formed by a Tn*7*-like transposon carrying accessory genes putatively involved in the degradation of certain aromatic compounds. Genomic islands formed by Tn*7*-like transposons have been identified in *H. chejuensis, S. sonnei *and *I. loihiensis*, in which Tn*7 *inserted into the *att*Tn*7 *site adjacent to a *glmS *gene [38]. In *C. metallidurans *CH34 this Tn*7*-like element is not found within the *att*Tn*7 *site and in addition lacks the *tnsE *gene and possesses two distinct *tnsD *genes. Parks and Peters [39] showed that the presence of two distinct *tnsD *genes is common in Tn*7*-like elements that are not found within the *att*Tn*7 *site and hypothesized that one of the TnsD proteins might actually allow non-specific target site recognition.

Both the CGH and physiological data indicated that the heavy metal resistance determinants identified in *C. metallidurans *CH34 are well conserved among other *C. metallidurans *strains. This strong conservation was also observed for genes encoding small stress responsive proteins and sigma factors, of which at least a part are involved in metal resistance [7,40].

The incidence of these metal resistance determinants could, however, not be directly related to their isolation source (biotype) nor location (geographic). This indicates that these resistance determinants are probably acquired earlier in evolution, which is consistent with the hypothesis that toxic metal resistance systems are preexistent to the recent anthropogenic activities and arose soon after life began, in a world already polluted by volcanic activities [41]. However, taking into account that most of the metal determinants are on the native megaplasmids and the GIs thereon, it could be argued that anthropogenic activities and technologized environments provided a selective pressure for the conservation of these determinants or even the acquisition of some, considering both the arsenal of determinants as well as the level of resistance to metals. Putatively, these determinants may even render a higher fitness in infections as these megaplasmids, despite their fitness cost, are also present in strains isolated from human infections. The structural and functional characteristics that metal resistance systems share with antibiotic resistance systems could be significant for this [42].

## Conclusions

Our comparative study showed that most metal resistance determinants identified in *C. metallidurans *CH34 are common to all *C. metallidurans *strains irrespective of the strain's isolation type and place. *C. metallidurans *strains do display considerable differences in the diversity and size of their mobile gene pool, which reflects at least some metabolic properties.

## Methods

### Strains, media and culture conditions

*C. metallidurans *strains used in this study are summarized in Table 1 and were routinely cultured at 30°C in Tris-buffered mineral medium (MM284) supplemented with 0.2% (wt/vol) gluconate as described previously [43]. Autotrophic growth on H_2 _and CO_2 _was scored by incubating on MM284 agar medium (without gluconate) under an atmosphere of H_2_/CO_2_/O_2 _(approximately 75:15:10; by vol.). Growth on toluene was scored by growing strains on MM284 agar medium (without gluconate) in a closed container saturated with toluene vapor. The maximum tolerable concentration (MTC), which is the highest tested concentration of a substance for which bacterial growth could be observed, was determined for ZnSO_4_, SrCl_2_, CoCl_2_, NiCl_2 _and K_2_CrO_4 _in liquid MM284. A stationary phase culture of each isolate was diluted 100-fold in liquid MM284 containing the metal concentration of interest. Cultures were incubated at 30°C and after 72 hours the MTC was scored. The MTC for Pb(NO_3_)_2 _was determined similarly except the analysis medium contained 0.4 g/L peptone, 0.4 g/L yeast extract, 0.4 g/L tryptone and 10 mM 2-N-morpholinoethanesulfonic acid to buffer the pH at 6.5 [adapted from [44]].

### Molecular analyses

Standard techniques were used for PCR and agarose gel electrophoresis. The oligonucleotides used for PCR were synthesized by Eurogentec (Seraing, Belgium) and are listed in Additional file 2: Table S5. Genomic DNA (gDNA) was prepared using the QIAamp DNA mini kit (Qiagen, Venlo, The Netherlands). Extraction of megaplasmids was based on the method of Andrup et al. [45].

### Genomic DNA labeling, array hybridization, washing and scanning

Four μg of gDNA was fragmented to 50 to 150 bp by partial digestion with *Sau*3AI (Fermentas, St. Leon-Rot, Germany). Next, gDNA was labeled with the BioPrime^® ^Array CGH Genomic Labeling System (Invitrogen, Merelbeke, Belgium). Labeled gDNA was re-suspended in the universal hybridization buffer of the Pronto kit (Promega, United States), mixed and added to the spotted slide (GEO accession number Platform GPL4980) for overnight hybridization at 42°C in a Tecan HS4800 Pro hybridization station (Tecan Group Ltd, Switzerland). Afterwards, slides were washed according to Pronto kit's protocol. Slides were scanned (at 532 and 635 nm) using the GenePix Personal 4100A microarray scanner (Molecular Devices^©^, USA).

### Array data and clustering analysis

Microarray spot-signals were analyzed using the GenePix Pro v.6.0.1 software and flagged according to build-in quality criteria. Raw median intensity data were imported into R version 2.13.1 for statistical analysis using the LIMMA package version 2.15.15 [46] as available from BioConductor. Raw data were background-corrected based on convolution of normal and exponential distributions with an offset of 50 [47]. Data were normalized within each array using the printing-tip loess normalization algorithm [48]. The microarray data have been deposited in the Gene Expression Omnibus website http://www.ncbi.nlm.nih.gov/geo/ under accession number GSE36303. Analysis of previously characterized *C. metallidurans *CH34 derivatives (loss of either pMOL28 or pMOL30 [5], IS*1071*-mediated loss of growth on CO_2 _and H_2 _[10]) allowed us to validate and optimize the trade-off between the number of false-positives and false-negatives. In the final analyses, a cut-off of 20-fold change in hybridization intensity compared to the background was used (Additional file 5: Table S6; GSE36303). Hierarchical clustering was performed using the complete linkage method with the hclust software from the R-package stats (version 2.13.1). Hierarchical clustering was obtained based on the pair-wise correlation between the different strains of the percentage of overlapping genes and percentage of GI genes conserved in Figures 1 and 2, respectively. Bootstrap values (n = 1000) were obtained using the R-package pvclust [49]. Heatmaps were produced using the heatmap.2 function as implemented in the R-package gplots (version 2.10.1). Gene annotations were retrieved from the up-to-date annotation of the different replicons of *C. metallidurans *CH34 available on GenoScope's MaGe system [50].

## Competing interests

The authors declare that they have no competing interests.

## Authors' contributions

PM performed the bioinformatics analyses. RVH wrote the manuscript. KM, AP and AJ provided experimental data. RVH, MM and NL provided the intellectual framework of the study and recommended analyses. All authors read and approved the final manuscript.

## Supplementary Material

Additional file 1 **Figure S1**. Plasmid patterns of *C. metallidurans *strains. Agarose gel electrophoresis of plasmid extracts from strains CH34 (1 and 18), KT01 (2), KT02 (3), KT21 (4), CH42 (5), CH79 (6), AS39 (7), AS167 (8), AS168 (9), 31A (10), SV661 (11), 43015 (12), 45957 (13), NE12 (14), NA1 (15), NA2 (16), and NA4 (17). Lower band represents chromosomal DNA (PDF 33 kb). Click here for file

Additional file 2 **Word document containing the Supplementary Tables 1 to 5 (DOC 146 kb)**. Click here for file

Additional file 3 **Figure S2**. Cartographic map of chromosome 2 of the different *C. metallidurans *strains. Negative hybridization signals are highlighted red. Newly identified putative genomic islands are indicated by blue bars (PDF 23 kb). Click here for file

Additional file 4 **Figure S3**. Cartographic map of chromosome 1 of the different *C. metallidurans *strains. Negative hybridization signals are highlighted red. Previously identified genomic islands are indicated by dark blue bars. Newly identified putative genomic islands are indicated by green bars (PDF 33 kb). Click here for file

Additional file 5 **Table S6**. (excel document) with the results of the comparative genomic hybridization (XLS 2902 kb). Click here for file
