# Supplementary figures and images for: Variation in genomic islands contribute to genome plasticity in cupriavidus metallidurans
Source: BMC Genomics. 2012 Mar 23;13:111. doi: 10.1186/1471-2164-13-111 (PMC3384475; doi:10.1186/1471-2164-13-111)

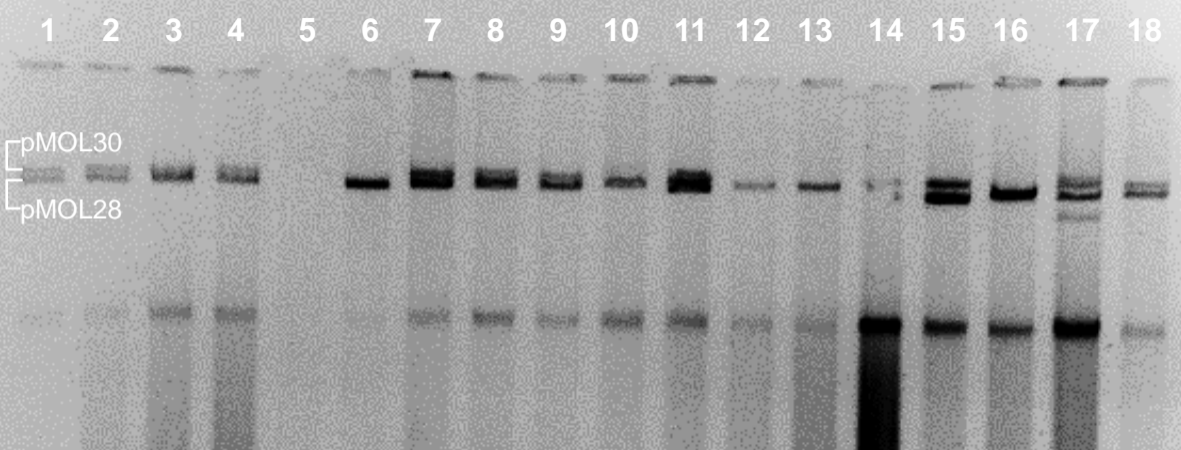

Supplement: Additional file 1 — Figure S1. Plasmid patterns of C. metallidurans strains. Agarose gel electrophoresis of plasmid extracts from strains CH34 (1 and 18), KT01 (2), KT02 (3), KT21 (4), CH42 (5), CH79 (6), AS39 (7), AS167 (8), AS168 (9), 31A (10), SV661 (11), 43015 (12), 45957 (13), NE12 (14), NA1 (15), NA2 (16), and NA4 (17). Lower band represents chromosomal DNA (PDF 33 kb). [file 1471-2164-13-111-S1.PDF]

# CHR2

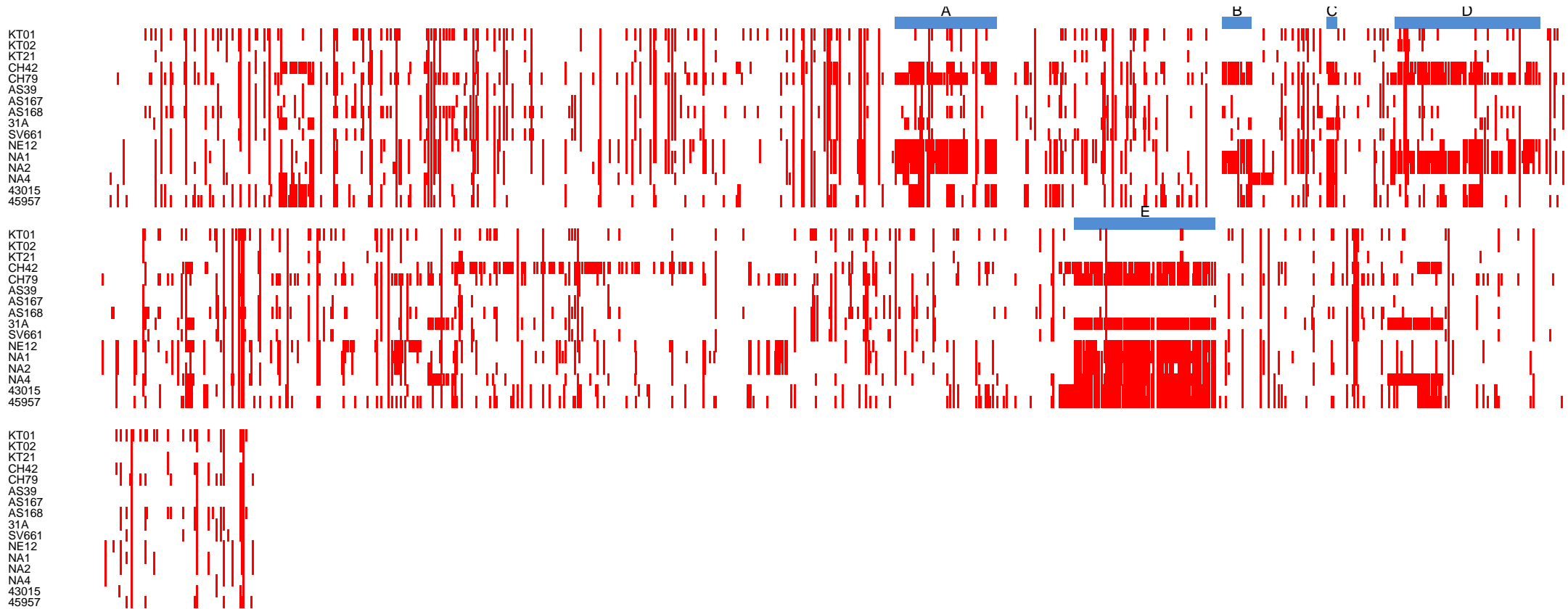

Supplement: Additional file 3 — Figure S2. Cartographic map of chromosome 2 of the different C. metallidurans strains. Negative hybridization signals are highlighted red. Newly identified putative genomic islands are indicated by blue bars (PDF 23 kb). [file 1471-2164-13-111-S3.PDF]

# CHR1

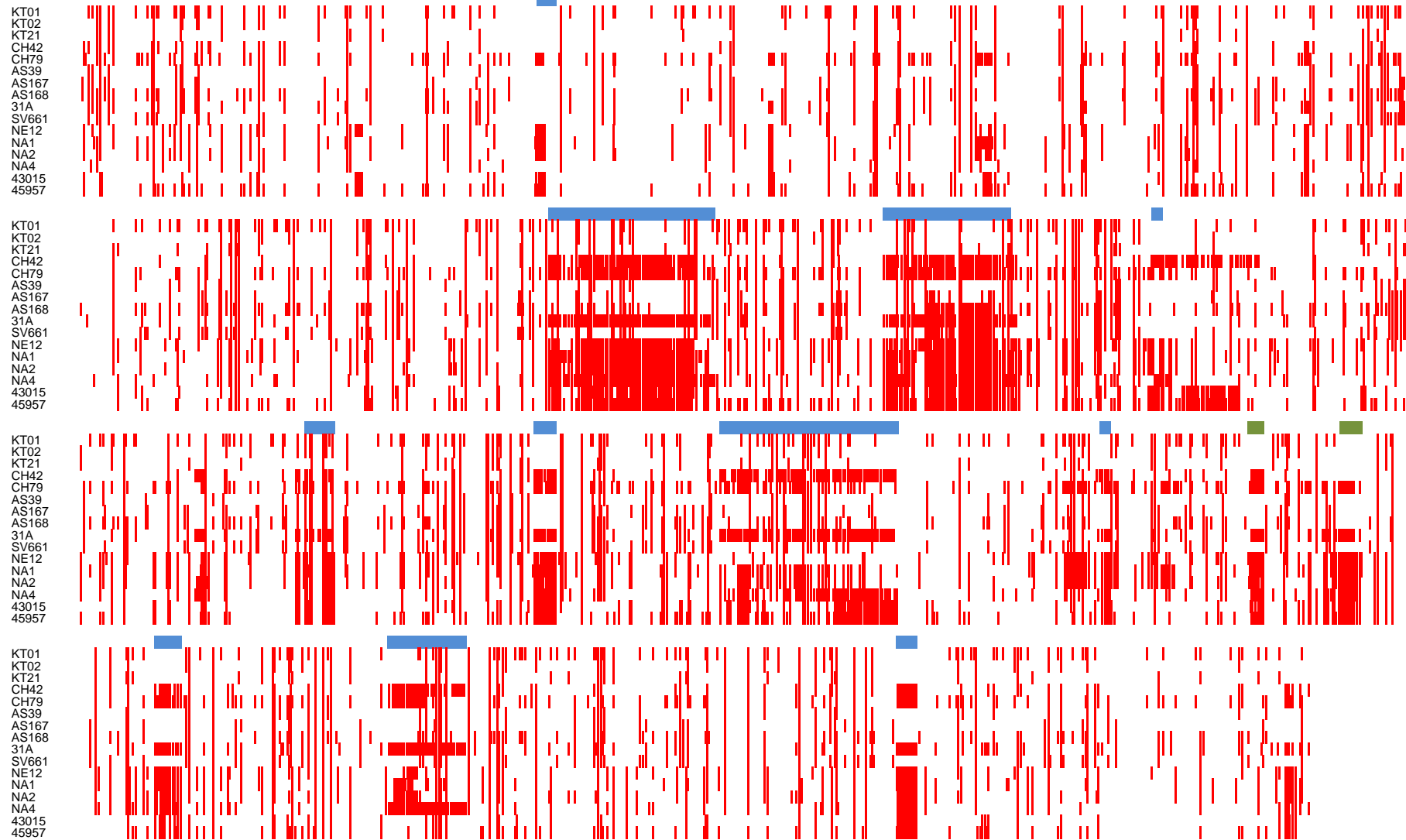

Supplement: Additional file 4 — Figure S3. Cartographic map of chromosome 1 of the different C. metallidurans strains. Negative hybridization signals are highlighted red. Previously identified genomic islands are indicated by dark blue bars. Newly identified putative genomic islands are indicated by green bars (PDF 33 kb). [file 1471-2164-13-111-S4.PDF]
