# Supplementary material for: Variation in genomic islands contribute to genome plasticity in cupriavidus metallidurans
Source: BMC Genomics. 2012 Mar 23;13:111. doi: 10.1186/1471-2164-13-111 (PMC3384475; doi:10.1186/1471-2164-13-111)
Supplement: Additional file 2 — Word document containing the Supplementary Tables 1 to 5 (DOC 146 kb). [file 1471-2164-13-111-S2.DOC]

| Supplementary Table 1. Relative occurrence of *C. metallidurans* CH34 genomic islands CMGI-2 and CMGI-3 and associated phenotypes in *C. metallidurans* strains. | | | | | | |
| --- | --- | --- | --- | --- | --- | --- |
| Strain | CMGI-2^a^ | AccMod^b^ | Toluene^c^ | CMGI-3^a^ | AccMod^b^ | H_2_ + CO_2_^c^ |
| AS39 | 86.6 | 86.1 | - | 89.5 | 93.6 | + |
| AS167 | 86.6 | 87.5 | + | 77.9 | 72.3 | + |
| AS168 | 78.6 | 77.8 | + | 47.7 | 21.3 | - |
| KT01 | 83.0 | 83.3 | + | 84.9 | 91.5 | + |
| KT02 | 86.6 | 86.1 | + | 91.9 | 95.7 | + |
| KT21 | 89.3 | 88.9 | + | 87.2 | 91.5 | + |
| SV661 | 83.0 | 83.3 | + | 51.2 | 23.4 | + |
| CH42 | 27.7 | 16.7 | - | 26.7 | 21.3 | - |
| CH79 | 22.3 | 12.5 | - | 22.1 | 17.0 | - |
| 31A | 15.2 | 8.3 | - | 20.9 | 17.0 | - |
| NE12 | 30.4 | 11.1 | + | 30.2 | 17.0 | - |
| NA1 | 18.8 | 8.3 | - | 26.7 | 17.0 | - |
| NA2 | 24.1 | 9.7 | - | 29.1 | 17.0 | - |
| NA4 | 12.5 | 8.3 | - | 20.9 | 14.9 | - |
| 43015 | 32.1 | 18.1 | ND | 30.2 | 17.0 | - |
| 45957 | 25.0 | 8.3 | ND | 24.4 | 10.6 | - |
| Percentage of genes related to the GI(^a^) or its accessory module (^b^) displaying a positive hybridization signal by CGH. ^c^Growth capability on toluene and H_2_ and CO_2_, respectively. | | | | | | |

| Supplementary Table 2. Maximum tolerable concentrations (mM) for tested metals. | | | | |
| --- | --- | --- | --- | --- |
|  | CrO_4_^2-^ | Ni^2+^ | Co^2+^ | Pb^2+^ |
| CH34 | 0.2 | 4 | 12.5 | 0.7 |
| AS39 | 0.4 | 4 | 12.5 | 0.7 |
| AS167 | 0.2 | 4 | 12.5 | 0.6 |
| AS168 | 0.2 | 4 | 12.5 | 0.6 |
| KT01 | 0.2 | 10 | 12.5 | 0.7 |
| KT02 | 0.2 | 40 | 12.5 | 0.6 |
| KT21 | 0.2 | 20 | 12.5 | 0.7 |
| SV661 | 0.2 | 4 | 12.5 | 0.6 |
| CH42 | 0.1 | 4 | 6.25 | 0.6 |
| CH79 | 0.1 | 4 | 12.5 | 0.7 |
| 31A | 0.4 | 40 | 12.5 | 0.7 |
| NE12 | 0.2 | 4 | 6.25 | 0.7 |
| NA1 | 0.2 | 3 | 6.25 | 0.7 |
| NA2 | 0.2 | 3.5 | 6.25 | 0.7 |
| NA4 | 0.2 | 20 | 6.25 | 0.6 |
| 43015 | 0.2 | >16 | 12.5 | 0.3 |
| 45957 | 0.2 | >16 | 12.5 | 0.3 |

|  | | | | | | | | | | | | | | | | | | |
| --- | --- | --- | --- | --- | --- | --- | --- | --- | --- | --- | --- | --- | --- | --- | --- | --- | --- | --- |
| Supplementary Table 3. Occurrence of *C. metallidurans* CH34 genes encoding sigma factors in *C. metallidurans* strains as indicated by CGH. | | | | | | | | | | | | | | | | | | |
| Gene | Replicon | CH34 locus tag | AS39 | AS167 | AS168 | KT01 | KT02 | KT21 | SV661 | CH42 | CH79 | 31A | NE12 | NA1 | NA2 | NA4 | 43015 | 45957 |
| *rpoH* | CHR1 | Rmet_0272 | + | + | + | + | + | + | + | + | + | + | + | + | + | + | + | + |
| *rpoN* | CHR1 | Rmet_0303 | + | + | + | + | + | + | + | + | + | + | + | + | + | + | + | + |
| *rpoO* | CHR1 | Rmet_0597 | + | + | + | + | + | + | + | + | + | + | + | + | + | + | + | + |
| *rpoR* | CHR1 | Rmet_0910 | - | - | - | - | + | + | - | + | - | - | + | + | + | + | + | + |
| *rpoI* | CHR1 | Rmet_1120 | + | + | + | + | + | + | + | + | + | + | + | + | + | + | + | + |
| *rpoP* | CHR1 | Rmet_1648 | + | + | + | + | + | + | + | + | **-** | + | **-** | **-** | - | + | - | - |
| *rpoS* | CHR1 | Rmet_2115 | + | + | + | + | + | + | + | + | + | + | + | + | + | + | + | + |
| *rpoE* | CHR1 | Rmet_2425 | + | + | + | + | + | + | + | + | + | + | + | + | + | + | + | + |
| *rpoD1* | CHR1 | Rmet_2606 | + | + | - | - | + | + | + | + | - | + | + | + | + | + | + | + |
| *rpoL* | CHR1 | Rmet_3280 | + | + | + | + | + | + | + | + | + | + | + | + | + | + | + | + |
| *fliA* | CHR2 | Rmet_3702 | + | + | + | + | + | + | + | + | + | + | + | + | + | + | + | + |
| *rpoT* | CHR2 | Rmet_3844 | + | + | + | + | + | + | + | + | + | + | + | + | + | + | + | + |
| *rpoK* | CHR2 | Rmet_4001 | + | + | + | + | + | + | + | + | + | + | + | + | + | + | + | + |
| *rpoJ* | CHR2 | Rmet_4499 | + | + | + | + | + | + | + | + | + | + | + | + | + | **-** | **+** | **+** |
| *rpoD2* | CHR2 | Rmet_4661 | + | + | + | + | + | + | + | + | + | + | **-** | **-** | + | + | + | - |
| *rpoQ* | CHR2 | Rmet_4686 | + | + | + | + | + | + | + | + | + | + | + | + | + | + | + | + |
| *rpoM* | CHR2 | Rmet_5400 | + | + | + | + | + | + | + | + | + | + | + | + | + | + | + | + |
| *cnrH* | pMOL28 | Rmet_6207 | - | - | - | - | - | + | - | - | - | + | - | - | - | + | + | + |

| Supplementary Table 4. Occurrence of *C. metallidurans* CH34 genes encoding small stress responsive proteins in *C. metallidurans* strains as indicated by CGH. | | | | | | | | | | | | | | | | | |
| --- | --- | --- | --- | --- | --- | --- | --- | --- | --- | --- | --- | --- | --- | --- | --- | --- | --- |
| CH34 locus tag | Gene | AS39 | AS167 | AS168 | KT01 | KT02 | KT21 | SV661 | CH42 | CH79 | 31A | NE12 | NA1 | NA2 | NA4 | 43015 | 45957 |
| Rmet_0477 |  | + | + | + | + | + | + | + | + | + | + | + | + | + | + | + | + |
| Rmet_1183 |  | + | + | + | + | + | + | + | + | + | + | + | + | + | + | + | + |
| Rmet_1797 |  | + | + | - | - | + | + | + | + | - | - | + | + | + | + | + | - |
| Rmet_3454 | *mmrQ_1_* | + | - | - | + | + | + | - | + | + | - | + | + | + | + | + | + |
| Rmet_3571 |  | + | + | + | + | + | + | + | + | + | + | + | + | + | + | + | + |
| Rmet_3641 |  | + | + | + | + | + | + | + | + | + | + | + | + | + | + | + | + |
| Rmet_3715 |  | + | + | + | + | + | + | + | + | + | + | + | + | + | + | + | + |
| Rmet_3909 |  | + | + | + | + | + | + | + | + | + | + | + | + | + | + | + | + |
| Rmet_4187 | *mmtQ* | + | + | + | + | + | + | + | + | + | + | + | + | + | + | + | + |
| Rmet_4264* |  |  |  |  |  |  |  |  |  |  |  |  |  |  |  |  |  |
| Rmet_4461 | *mmmQ* | + | + | + | + | + | + | + | + | + | + | + | + | + | + | + | + |
| Rmet_4908^#^ | *mmsQ_1_* | + | + | + | + | + | + | + | + | + | + | + | + | + | + | + | + |
| Rmet_5281 |  | + | + | + | + | + | + | + | + | + | + | + | + | + | + | + | + |
| Rmet_5594^#^ | *mmsQ_2_* | + | + | + | + | + | + | + | + | + | + | + | + | + | + | + | + |
| Rmet_5620 |  | + | + | + | + | + | + | + | + | + | + | + | + | + | + | + | + |
| Rmet_5975 | *czcJ* | + | + | + | + | + | + | + | + | + | + | + | + | + | + | + | + |
| Rmet_6121 | *copQ* | + | + | + | + | + | + | + | + | + | + | + | + | + | + | + | + |
| Rmet_6124* | *copW* |  |  |  |  |  |  |  |  |  |  |  |  |  |  |  |  |
| Rmet_6143 | *mmrQ_2_* | + | + | + | + | + | + | + | - | + | + | + | + | + | + | + | - |
| *No oligonucleotide probe available on microarray, ^#^identical genes. | | | | | | | | | | | | | | | | | |

| Supplementary Table 5. PCR primers used in this study. | | |
| --- | --- | --- |
| Primer name | Sequence (5' 🡪 3') | Primer annealing coordinates |
| hmyB_FW | GAAGGGCAGAGCGTCGATATC | 692049-692069 (CHR2) |
| hmyA_RV | GCATAGCGCACCGAATTGA | 696246-696264 (CHR2) |
| mmmQ_FW | AGTCTCTAGAACGCGAGCTACTTCTTCGAG | 1058076-1058097 (CHR2) |
| czcR2i_RV | CTAGAAGCTTGGTGTTGCTGTCGAAGTTCA | 1062197-1062218 (CHR2) |
| czcA1(c711)_FW | CGTGGTCTACGGTTTCACG | 2438423-2438441 (CHR2) |
| czcA2(c711)_RV | CACACCGTTGATCTTGCGTA | 2436991-2437010 (CHR2) |
| czcC2 | TGTACACAGCCATCCGTCAG | 1217038-1217057 (CHR2) |
| czcA2R | GTACGAGGCCAGCTTTTCAG | 1066866-1066885 (CHR2) |
| pbrUa_FW | GTCTTCTGGGTGGCAGTCAT | 117837-117856 (pMOL30) |
| pbrUb_RV | GCACCTGCATAGAACGTGAA | 121954-121973 (pMOL30) |
|  |  |  |
